# Low-Energy Traumatic Obturator Hip Dislocation with Ipsilateral Femoral Shaft Fracture in a Patient with Omolateral Knee Arthroplasty

**DOI:** 10.1155/2016/8754893

**Published:** 2016-10-17

**Authors:** G. Gazzotti, L. Patrizio, S. Dall'Aglio, E. Sabetta

**Affiliations:** ^1^Unit of Orthopedic Surgery, IRCCS-Arcispedale Santa Maria Nuova, Reggio Emilia, Italy; ^2^Unit of Orthopedic Surgery, Ospedale Santa Maria dello Splendore, Giulianova, Teramo, Italy

## Abstract

Ipsilateral obturator hip dislocation and femoral shaft fracture are rare. We report such a case in an older woman after a low-energy injury. She had a knee prostheses in the same limb. The patient was treated by open manipulative reduction of the luxation without opening joint and open reduction and internal fixation of the femur with angular stability plate and screws. We could not find a similar case in the literature. An early diagnosis of the dislocation is crucial in order to obtain good results. Great awareness and radiologic examination are fundamental to achieve precocious diagnosis of both these rare combined injuries, as treatment in these cases is considered an emergency. The first step was an attempt to reduce the dislocation by closed means but it failed. Then we performed a short approach at the trochanteric region and used Lambotte forceps to manoeuvre the proximal femur without opening the joint achieving reduction. Thereafter the femoral shaft fracture underwent open reduction and internal fixation with an angular stable plate. After a 2-year follow-up the outcome was very good.

## 1. Introduction

Anterior hip dislocation with associated ipsilateral femoral shaft fracture is a very rare injury, with few cases reported in the literature. They are generally related to high-energy trauma (sport or traffic accidents) in young people. We report a rare low-energy injury in an elderly woman: obturator hip dislocation associated with ipsilateral femur shaft fracture in a patient with knee arthroplasty. She was treated by open manipulative reduction of the dislocation reduction without opening joint using Lambotte forceps and open reduction and internal fixation of femur with angular stability plate and screws.

## 2. Case Report

**Figure 1:**
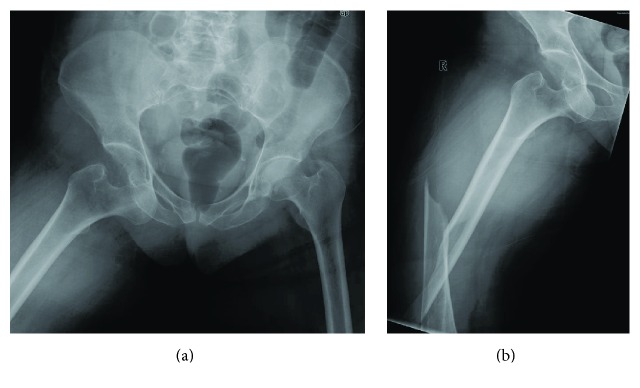
Preoperative X-ray examination.

**Figure 2:**
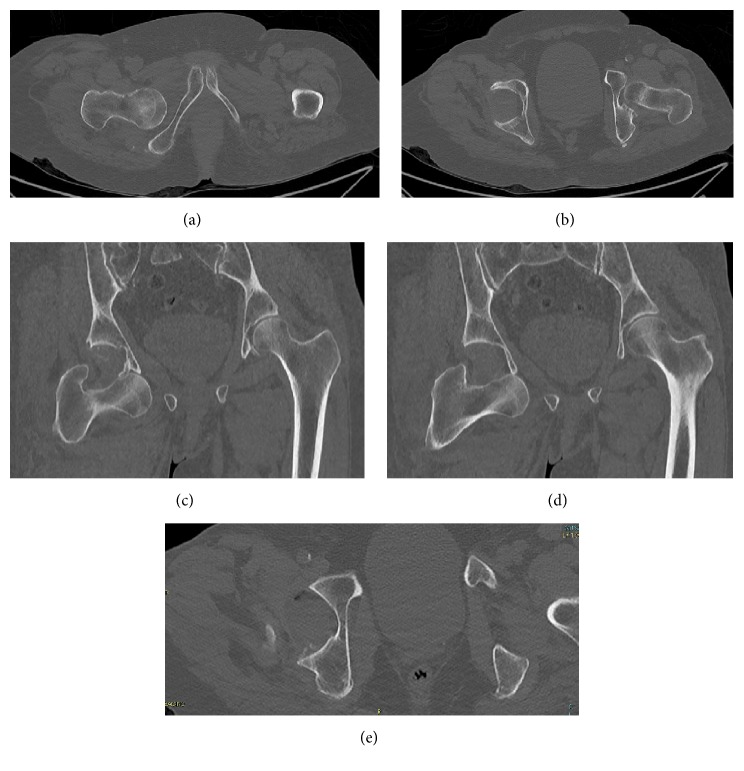
Preoperative CT scan.

**Figure 3:**
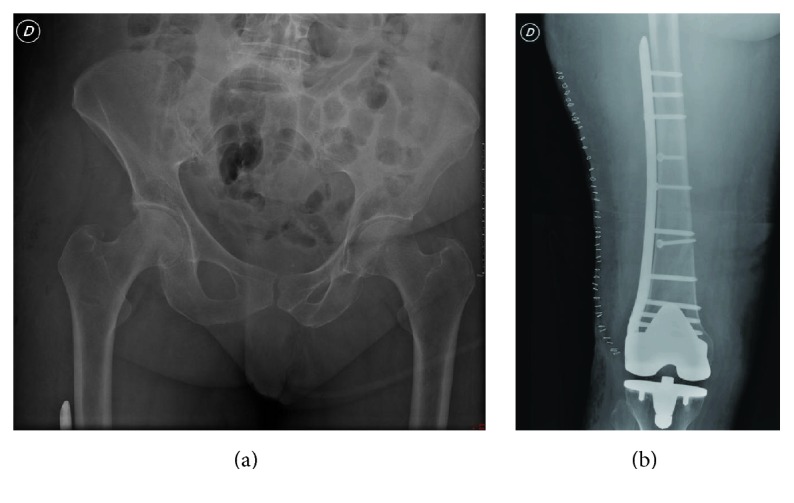
Postoperative X-ray examination.

**Figure 4:**
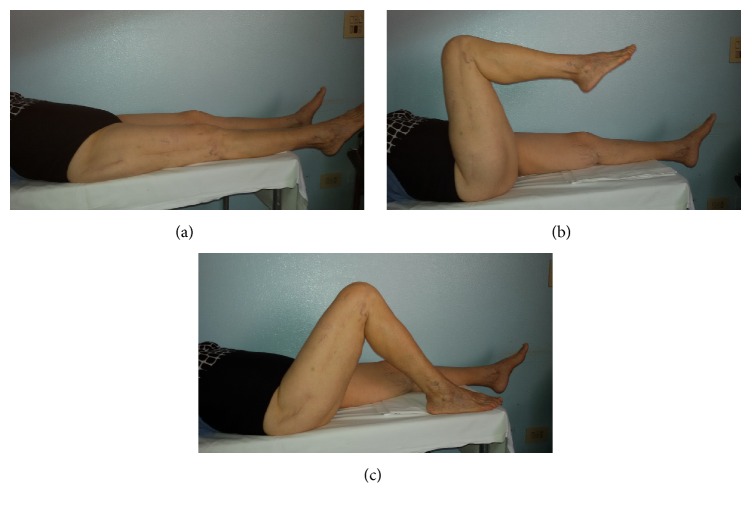
Clinical evaluation at 2-year follow-up.

**Figure 5:**
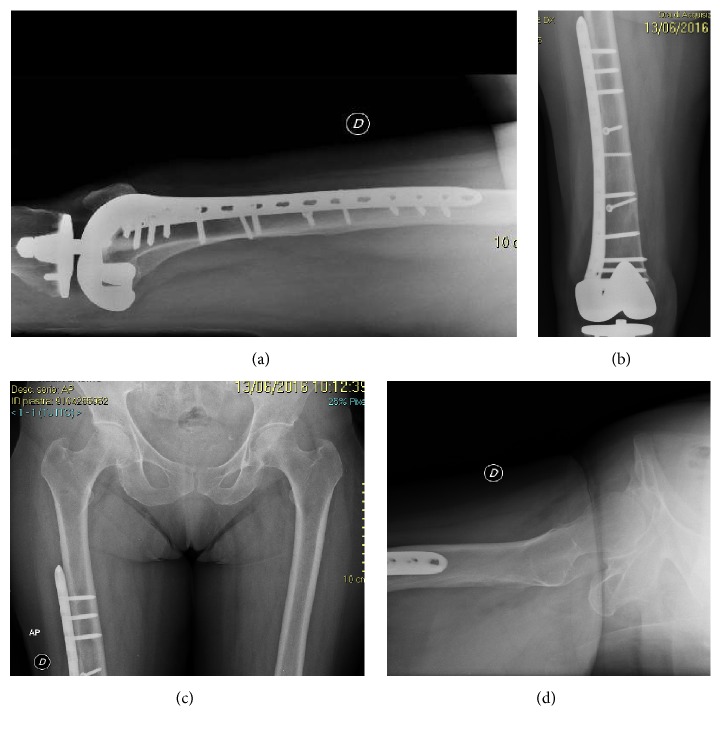
X-ray examination at 2-year follow-up.

A 78-year-old woman, while on holiday at the seaside, stumbled as she was pushing her son's wheelchair and fell down a flight of stairs in a hotel. She was transferred to the Emergency Room of the local Hospital. Radiologic assessment revealed an obturator hip dislocation with ipsilateral femur shaft fracture in this patient with knee arthroplasty (Figures 1(a)-1(b)). The patient refused treatment in this facility and was transferred to our Hospital (Hospital Arcispedale Santa Maria Nuova) in Reggio Emilia after a three-hour trip. We evaluated the patient 6 hours after trauma. During clinical examination, we noted an anterior hematoma in the proximal thigh, the hip in adduction and external rotation, and the knee and leg in complete external rotation. Neurological and vascular examinations were normal. A CT scan was performed in order to provide further assessment of injuries in order to help the surgical planning (Figures 2(a)-2(b)). No femoral head fracture was present (Figures 2(c)-2(d)), and in the posterior-inferior rim of the acetabular cavity small calcified images compatible with millimetric free fragments were detected (Figure 2(e)). A surgical repair of every lesion was proposed. First, a closed reduction of hip dislocation under general anaesthetic was attempted but the femoral shaft fracture made reduction impossible. A small incision was made under the base of the greater trochanter and manipulation of the proximal femur with a Lambotte forceps without opening the hip joint was successful in reducing the dislocation. A second larger, distal, and lateral incision was made to reduce and fix the femoral shaft fracture with interfragmentary screws and an angular stable plate under fluoroscopic guide (Figures 3(a)-3(b)). During the postoperative period, the patient carried out active and passive mobilization of the hip and knee without bearing weight for 4 weeks. Then she started walking with crutches, bearing partial weight (20 kg) on the injured limb for 2 weeks, increasing 15 kg per week. After 45 days, she was able to walk with full weight-bearing. Five months after the injury the hip had full range of pain-free motion, there was no limb shortening, and radiographs confirmed shaft fracture healing. At 12 months, she returned on holiday at the seaside with her son. At the 2-year follow-up, there was complete painless hip function (Figures 4(a)–4(c)) and radiographs did not show any evidence of avascular necrosis (Figures 5(a)–5(d)).

## 3. Discussion

Hip dislocation associated with femoral shaft fracture is a rare condition [1]. Wiltenberger et al. estimated hip dislocation incidence at 1 in 100000 femur shaft fractures [2]. A literature review of these combined injuries indicates that the hip dislocation is primarily missed in nearly half of the cases, and this is for different reasons [3–5]. First, it is an uncommon combined injury. Second, it is generally associated with severe shock and in the first aid, more attention is paid to resuscitation. Third, shaft fractures often obscure clinical signs of the dislocated hip. A sign that should be searched and might be for diagnosis in an anterior hip dislocation is the palpation of the femoral head in the midinguinal region as well as a less prominent greater trochanter [6]. Generally, these combined injuries occur in young and middle age adults and are caused by high-energy trauma due to sport, pedestrian, or traffic accidents [1]. We could not find low-energy traumas causing this type of injury in the world literature. Helal and Skevis [7] postulated that the trauma mechanism in a similar case was related to two separate forces: an axial force on the flexed femur which causes the dislocation and a direct trauma, subsequent to the fall, which causes the shaft fracture. The degree of abduction or adduction of the thigh determines whether the dislocation is anterior or posterior. Most hip dislocations are posterior [8]; anterior hip dislocation is less common and is of two main types: superior (in the iliac or pubic region) or inferior (obturator region). Delayed reduction of hip dislocation is more difficult to obtain and it is related to several complications such as avascular necrosis of femoral head [9]. Reduction, even in acutely diagnosed cases, is extremely difficult because the femur, that is, the lever, is not intact and therefore it does not provide for control on the proximal femur [10, 11]. Therefore, closed manipulation may be attempted but it is effective in less than 50% of the cases [3, 6, 12, 13]. Lyddon and Hartman [14] described a method of closed reduction using a device on the trochanter to facilitate manipulation. Ingram and Turner [15] used a Steinman pin drilled through the greater trochanter. Helal and Skevis [7] suggested using a traction screw into the femoral neck or an intramedullary Denness' device in the proximal shaft of the femur to obtain a better leverage and make reduction easier. Also Dehne and Immermann [3] suggested an operative exposure of the fracture site and direct manipulation of the proximal fragment. Open reduction of the hip is recommended if previously described closed methods would fail, although this is associated with an increased risk of avascular necrosis of the femoral head [16]. Watson-Jones recommended ORIF of the femoral shaft fracture followed by closed manipulative reduction of hip [17]. Also Sambandan [18] preferred to treat the fracture first. He reported a case report of a twenty-year-old male who sustained a motor vehicle accident with associated injuries and cerebral concussion. He described an obturator dislocation associated with a femur shaft fracture managed by internal fixation of the femur with a Kuntscher nail followed by closed manipulative reduction of hip.

In the case reported here, at the latest follow-up examination (2 years), the patient had full range of passive motion with no pain or disability. Schoenecker et al. [12] reported three cases; one of them was an 18-year-old man involved in a motor vehicle accident with an anterior hip dislocation with ipsilateral femoral shaft fracture. The dislocation was reduced by closed means using a large bone clamp and the fracture was reduced and fixed with an intramedullary rod. At one-year postinjury, the patient had excellent results with no discomfort and radiologic assessment revealed that the fracture had healed and the hip joint appeared normal. We used a similar technique to reduce the hip dislocation without opening the joint even if we agree that closed manipulations might be attempted first. In our case, the shaft fracture was spiroid and we preferred to use interfragmentary screws and an angular stable plate in order to fix the shaft fracture.
